# Supplementary material for: Novel Applications of Lactic Acid and Acetic Acid Bacteria Preparations in Shaping the Technological and Microbiological Quality of Ready-to-Cook Minced Pork
Source: Foods. 2025 May 29;14(11):1934. doi: 10.3390/foods14111934 (PMC12154357; doi:10.3390/foods14111934)
Supplement: Supplementary file 1 [file foods-14-01934-s001.zip › foods-3645590-supplementary.pdf]

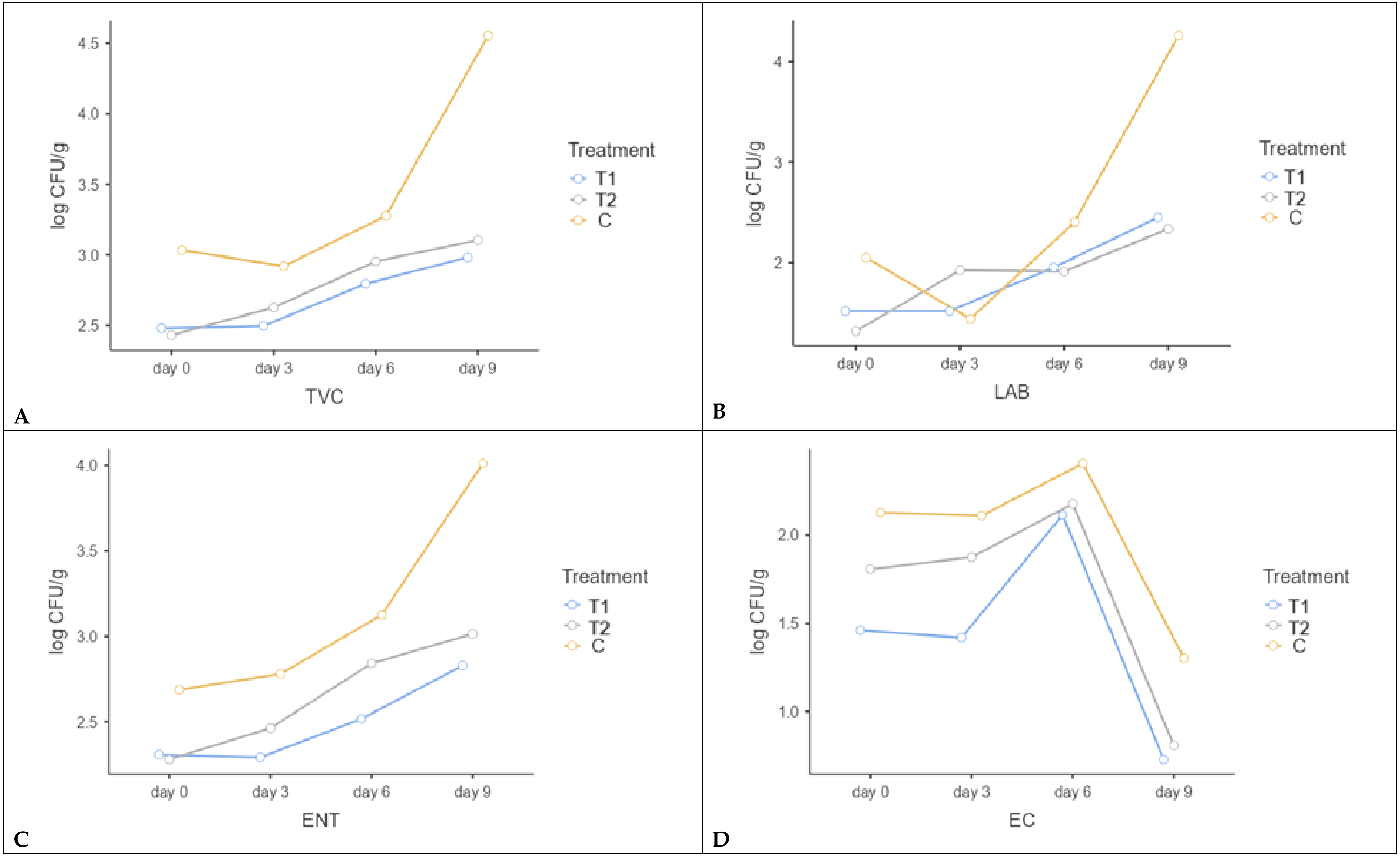

Figure S1. Growth of tested microorganisms in ready-to-cook minced meat samples during storage; A—TVC, total viable count; B—LAB, lactic acid bacteria; C—ENT, bacteria Enterobacteriaceae family; D—EC, *Escherichia coli*;

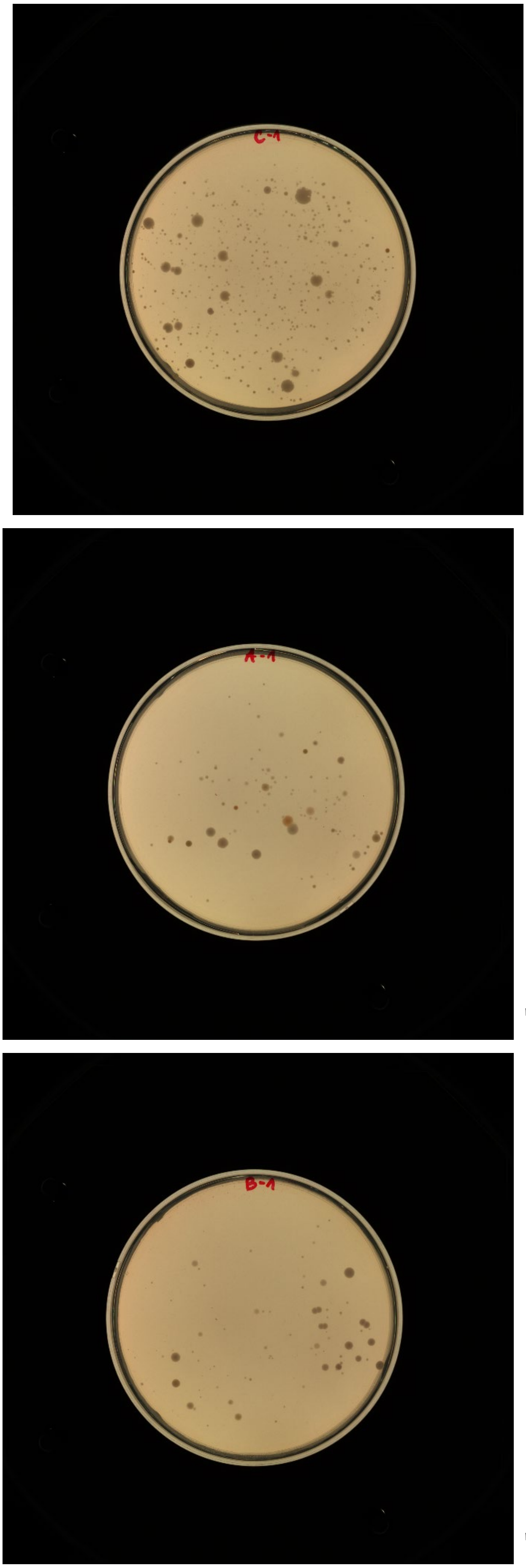

C

T1

T2

Figure S2. The photos of TVC agar plates after incubation at a given temperature and time for the three tested samples from one of the storage periods; C—control sample; T1—sample with the addition hkCFS of *Lacticaseibacillus paracasei* B1 and CFS of *Komagataeibacter saccharivorans* KOM1; T2—sample with the addition hkCFS of *Lactiplantibacillus plantarum* O24 and CFS of *Gluconobacter oxydans* KNS32

Table S1. Correlations between factors for day 0 of storage

	1	2	3
**1**	—	-0.025	0.147
**2**		—	0.167
**3**			—

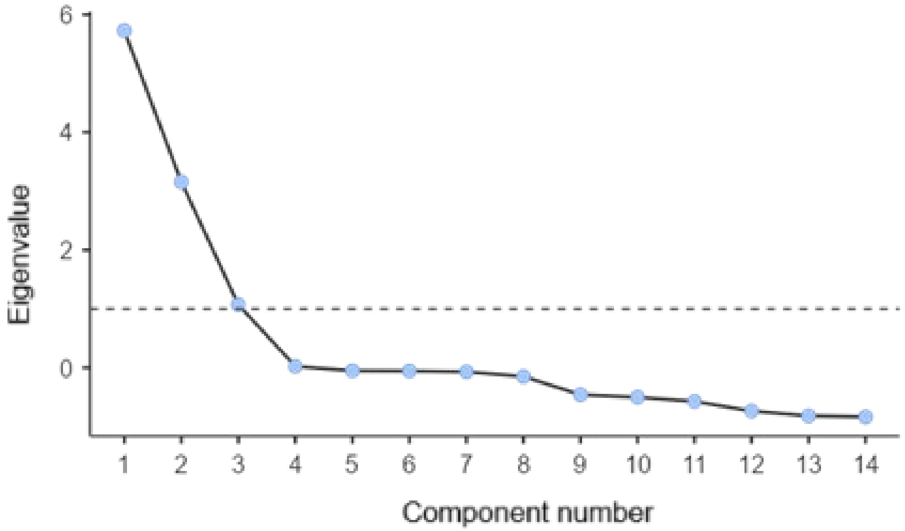

Figure S3. Scree plot for the components on day 0 of storage

Table S2. Correlations between factors on day 9 of storage

	1	2	3
**1**	—	0.139	-0.039
**2**		—	-0.128
**3**			—

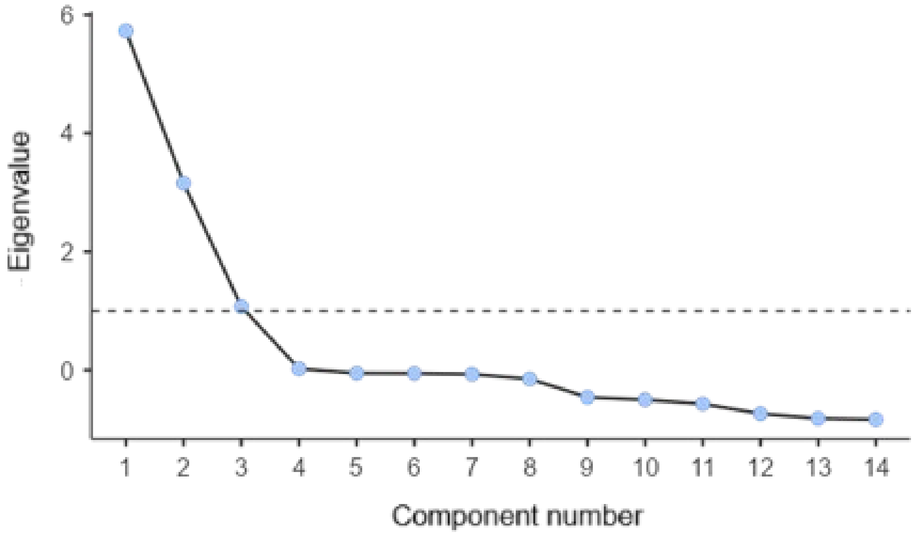

Figure S4. Scree plot for the components on day 9 of storage
